# Prevalence of Anxiety, Depression, and Perceived Stigma in Healthcare Workers in Nepal During Later Phase of First Wave of COVID-19 Pandemic: A Web-Based Cross-Sectional Survey

**DOI:** 10.7759/cureus.16037

**Published:** 2021-06-29

**Authors:** Suman P Adhikari, Namrata Rawal, Dhan B Shrestha, Pravash Budhathoki, Sabin Banmala, Shila Awal, Ganesh Bhandari, Rajesh Poudel, Avishek R Parajuli

**Affiliations:** 1 Department of Neuro-Psychiatry, Nepalese Army Institute of Health Sciences, Kathmandu, NPL; 2 Department of Internal Medicine, Mount Sinai Hospital, Chicago, USA; 3 Department of Internal Medicine, BronxCare Health System, Bronx, USA; 4 Department of Internal Medicine, Nepalese Army Institute of Health Sciences, Kathmandu, NPL; 5 Department of Community Medicine, Nepalese Army Institute of Health Sciences, Kathmandu, NPL

**Keywords:** anxiety, covid-19, depression, health personnel, nepal

## Abstract

Introduction

The COVID-19 pandemic has caused discrimination and social stigma among healthcare workers (HCW) causing psychological problems due to prolonged work shifts, uncertain pay, lack of personal protective equipment (PPE), added fear of infection to self or family, and so on. This online survey is directed towards the determination of anxiety, depression, and stigma among healthcare providers in Nepal during the later phase of the first wave of the COVID-19 pandemic.

Materials and methods

Anxiety and depression were assessed using standard Generalized Anxiety Disorder-7 (GAD-7), and Patient Health Questionnaire-9 (PHQ-9), respectively. Data for the survey were collected from January 10, 2021, to February 6, 2021, and analyzed using Stata 15 (College Station, TX: StataCorp LLC).

Results

A total of 213 participants were enrolled in the study from different parts of Nepal and their mean age was 29.90±6.43 years. The prevalence of anxiety and depression among healthcare workers was 46.95% and 41.31%, respectively. A bidirectional relationship was present between GAD-7 and PHQ-9 score interpretation. About 57% of HCW experienced some form of perceived stigmatization due to COVID-19. Frontline HCW were six times more likely to be stigmatized compared to non-front line HCWs and diagnosis of COVID-19 was associated with three times higher odds of facing perceived stigmatization.

Conclusion

A significant number of HCW experienced symptoms of anxiety and depression during the later phase of the COVID-19 pandemic. Frontline HCW who were infected experienced a higher level of stigma.

## Introduction

The outbreak of several cases of viral pneumonia occurred in January 2020 in Wuhan of China which spread globally and was declared a pandemic by WHO later on March 1 [1,2]. Nepal reported its first case of COVID-19 on January 23 and the first mortality on May 27. As of November 11, 2020, there are 202,329 confirmed cases and 1,174 deaths including deaths of HCWs [3]. With the increasing number of COVID-19 cases and mortality, frontline HCWs are under extreme conditions of discrimination and social stigma from both the community and other healthcare workers (HCWs) who are involved in non-COVID responses putting them at a higher risk of psychological problems [4].

In a cross-sectional study conducted in China, the prevalence of symptoms of anxiety, depression, insomnia, and the overall psychological problems in HCWs during the COVID-19 pandemic were 46.04%, 44.37%, 28.75%, and 56.59%, respectively [5]. In a study conducted in Nepal from April to June, 41.9%, 37.5%, and 33.9% of HCWs developed symptoms of anxiety, depression, and insomnia, respectively, with stigma shown to be significantly associated with these conditions [6].

The COVID-19 pandemic has been a life-changing experience for almost all people around the world. HCWs are also facing the pressure of working, especially in resource-poor settings. The psychological stress among the HCWs is attributed to prolonged work shifts, uncertain pay, lack of personal protective equipment (PPE), added fear of infection to self or family, having to stay in quarantine, as well as feelings of being inadequately supported in the workplace [6,7].

The impact of the COVID-19 pandemic on the mental health of healthcare workers is well documented in various countries but only a little information is available in the context of Nepal. Few studies were conducted during the early phase of the pandemic, and thus, the mental health outcomes might still reflect conditions existing before the pandemic [6,7]. This is a nationwide survey of the prevalence of anxiety, depression, and stigma in the HCWs of Nepal including doctors, nurses, health assistants, community health workers, health assistants, and other support staff during the later phase of the first wave of the COVID-19 pandemic.

## Materials and methods

This is a nationwide survey of the prevalence of anxiety and depression in the HCWs of Nepal including doctors, nurses, health assistants, community health workers, health assistants, and other support staff during the later phase of the first wave of the COVID-19 pandemic. The survey data were collected through an online Google form with informed consent from January 10, 2021, to February 6, 2021. A structured Google form was published in social media networks and sent in personal mail and messages requesting participants to share the survey form with other HCWs. Single response from each participant was ensured via Google form setting by choosing ‘Limit to a single response’ and later checked during manual data checks. The inclusion criteria were health workers aged 18 years and above working in Nepal. Participants were excluded if they were below 18 years of age, on leave, or unable to participate due to personal circumstances.

Anxiety and depression were assessed using Generalized Anxiety Disorder-7 (GAD-7), and Patient Health Questionnaire-9 (PHQ-9) [8,9]. GAD-7 is a self-administered patient questionnaire, used as a screening tool and severity measure for GAD consisting of seven items each of which is scored 0-3. The total scores of 5, 10, and 15 are taken as the cut-off points for mild, moderate, and severe anxiety, respectively. Similarly, the score of the PHQ-9 scale which measures the depression in the past two weeks was categorized as minimal (0-4), mild (5-9), moderate (10-14), moderately severe (15-19), and severe (20-27).

The dependent variables in the study included the status of perceived stigma by individuals, anxiety, and depression. Stigma is defined as a mark of disgrace associated with a particular circumstance, quality, or person. In Nepal, different forms of stigmatization like denied lodging and fooding were observed, if those HCWs were working as frontline workers in COVID dedicated units. The independent variables included information about demographic details like age, gender, province of residency, marital status, education, comorbidities, etc., and work-related variables like work experience, working as a frontline worker (defined as health workers working in COVID-19 dedicated sections), precautionary measures (perceived as sufficiently protected if getting standard PPE), etc. (detailed in tables in result section).

Sample size

The sample size was calculated with standard Cochran’s Sample Size Formula taking a proportion of psychological distress of 11.5% from a prior study among Nepalese residents [10].

X = Z^2^[p̂{(1- p̂) / ε^2^}]

Where z is the z score, ε is the margin of error, p̂ is the population proportion.

The calculated sample size was 157 considering the level of confidence as 95% with a 5% margin of error. Considering a 10% margin of non-response rate, the adjusted sample size was 173.

Research ethics

All respondents gave their informed consent for inclusion before they participated in the study. The study was conducted following the protocol and approved by the Ethical Review Committee of the Nepalese Army Institute of Health Sciences (NAIHS; Reference no: 367).

Statistical analysis

The data obtained in the study were exported in Excel and data were cleaned. Then data were imported and analyzed in STATA version 15 (College Station, TX: StataCorp LLC). Simple descriptive and cross-tabulation of various studied variables were done about anxiety, depression, and stigma. Chi-square test and Fisher exact test were performed to evaluate the association among the categorical variables considering 5% standard error and p-value cut off of 0.05 as a level of significance. Logistic regression (binary and multinomial) was performed to estimate unadjusted and adjusted odds ratio taking independent variables among which the chi-square test showed an association. A Scatter plot was drawn among continuous variables (GAD-7 total score vs. age and PHQ-9 score vs. age) to check the correlation.

## Results

Among a total of 215 responses, two forms were incomplete and thus excluded from the analysis.

Anxiety, depression, and stigma among healthcare practitioners during COVID-19

**Figure 1:**
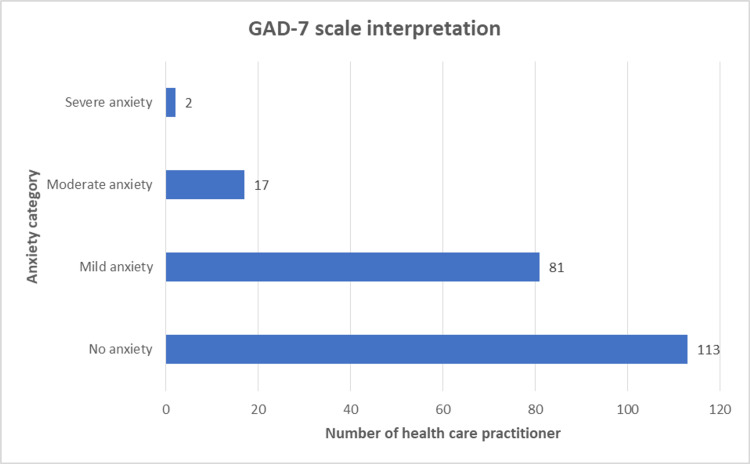
GAD-7 scale-based classification of healthcare practitioners

Among 213 complete responses, 53.05 % (n=113) were classified as no anxiety (GAD-7 score <5); while rest 46.95% (n=100) with some extent of anxiety [38.03%, mild anxiety (GAD-7 score 5-9); 7.98%, with moderate anxiety (GAD-7 score 10-14); 0.94% with severe anxiety (GAD-7 score >15)] (Figure 1).

Chi-square or Fisher’s exact test was applied to check the association among categorical independent variables with observed dependent variables. Among categorical independent variables observed, age category, gender, education level, stigma due to COVID-19, work experience, hospital admission due to COVID-19, PHQ-9 interpretation for depression, and health practitioner category were associated with anxiety category based on GAD-7 interpretation (p<0.05; Table 1).

**Table 1:** Cross-tabulation of independent variables across anxiety category using Chi-square test *Fisher’s exact test employed.

Variables		GAD-7 based diagnosis of anxiety			p-Value
		No, n(%)	Yes, n(%)	Total, n(%)	
Age (in years)	Less than 30	55(45.45)	66(54.55)	121(100.00)	0.011
	30 and above	58(63.04)	34(36.96)	92(100.00)	
	Mean±SD, 29.90±6.43; median, 28; range, 19-55				
Gender	Female	47(43.93)	60(56.07)	107(100.00)	0.007
	Male	66(62.26)	40(37.74)	106(100.00)	
Type of health institute	Government	55(56.12)	43(43.88)	98(100.00)	0.407
	Private	58(50.43)	57(49.57)	115(100.00)	
Province	Bagmati Province	71(50.00)	71(50.00)	142(100.00)	0.135*
	Gandaki Province	4(57.14)	3(42.86)	7(100.00)	
	Karnali Province	4(100.00)	0(0.00)	4(100.00)	
	Lumbini Province	6(46.15)	7(53.85)	13(100.00)	
	Province no. 2 (Janakpur as territorial capital)	6(50.00)	6(50.00)	12(100.00)	
	Province no. 1 (Biratnagar as territorial capital)	13(81.25)	3(18.75)	16(100.00)	
	Sudurpaschim Province	9(47.37)	10(52.63)	19(100.00)	
Education	Bachelor	50(45.45)	60(54.55)	110(100.00)	0.031*
	Intermediate	11(45.83)	13(54.17)	24(100.00)	
	Masters or above	48(64.86)	26(35.14)	74(100.00)	
	Secondary school level	4(80.00)	1(20.00)	5(100.00)	
Marital Status	Married	54(55.10)	44(44.90)	98(100.00)	0.580
	Single	59(51.30)	56(48.70)	115(100.00)	
Chronic diseases	No	105(52.76)	94(47.24)	199(100.00)	0.751
	Yes	8(57.14)	6(42.86)	14(100.00)	
History of psychiatric illness	No	110(54.19)	93(45.81)	203(100.00)	0.195*
	Yes	3(30.00)	7(70.00)	10(100.00)	
Medication for psychiatric illness	No	108(54.82)	89(45.18)	197(100.00)	0.069
	Yes	5 (31.25)	11(68.75)	16(100.00)	
Psychiatric support in pandemic	No	103(54.50)	86(45.50)	189(100.00)	0.235
	Yes	10(41.67)	14(58.33)	24(100.00)	
Chronic diseases in family members	No	51(53.13)	45(46.88)	96(100.00)	0.984
	Yes	62(52.99)	55(47.01)	117(100.00)	
Living with the elderly (>60yrs)	No	56(55.45)	45(44.55)	101(100.00)	0.506
	Yes	57(50.89)	55(49.11)	112(100.00)	
Frontline worker	No	27(61.36)	17(38.64)	44(100.00)	0.215
	Yes	86(50.89)	83(49.11)	169(100.00)	
Perceived stigma due to COVID-19	No	54(60.67)	35(39.33)	89(100.00)	0.045
	Yes	54(46.55)	62(53.45)	116(100.00)	
Work experience	Less than five years	61(47.29)	68(52.71)	129(100.00)	0.037
	More than five years	52(61.90)	32(38.10)	84(100.00)	
Precautionary measures	Insufficient	78(55.32)	63(44.68)	141(100.00)	0.353
	Sufficient	35(48.61)	37(51.39)	72(100.00)	
COVID-19 diagnosed	No	90(55.56)	72(44.44)	162(100.00)	0.192
	Yes	23(45.10)	28(54.90)	51(100.00)	
Admitted to the hospital due to COVID-19?	No	112(54.63)	93(45.37)	205(100.00)	0.027*
	Yes	1(12.50)	7(87.50)	8(100.00)	
Lost a significant one due to COVID-19	No	105(55.26)	85(44.74)	190(100.00)	0.063
	Yes	8(34.78)	15(65.22)	23(100.00)	
PHQ-9 interpretation	No depression	105(84.00)	20(16.00)	125(100.00)	0.000*
	Mild depression	8(11.94)	59(88.06)	67(100.00)	
	Moderate depression	0(0.00)	17(100.00)	17(100.00)	
	Moderately severe depression	0(0.00)	3(100.00)	3(100.00)	
	Severe depression	0(0.00)	1(100.00)	1(100.00)	
Depression	No	105(84.00)	20(16.00)	125(100.00)	0.000
	Yes	8(9.09)	80(90.91)	88(100.00)	
Healthcare workers	Other than doctor	42(44.68)	52(55.32)	94(100.00)	0.030
	Doctor	71(59.66)	48(40.34)	119(100.00)	

**Figure 2:**
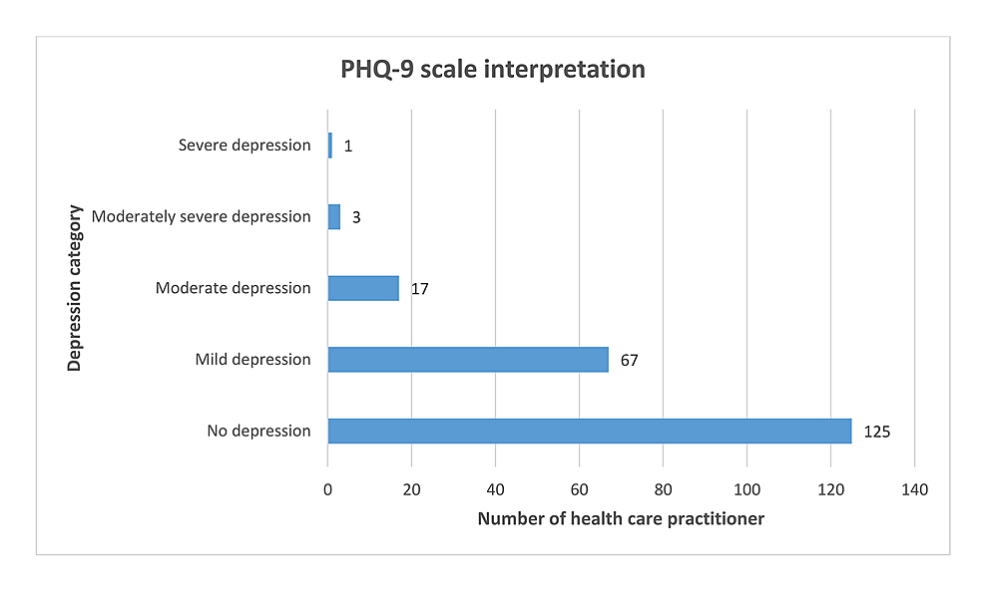
PHQ-9 scale-based classification of healthcare practitioners

Among 213 respondents, 58.69% (n=125) were classified as having no depression (PHQ-9 score <5); while rest 41.31% (n=88) had some extent of depression; 31.46%, of participants had mild depression (PHQ-9 score 5-9); 7.98% had moderate depression (PHQ-9 score 10-14); 1.41% had moderately severe depression (PHQ-9 score 15-19); and 0.47% had severe depression (PHQ-9 score >20) (Figure 2).

Among categorical independent variables observed, age category, gender, stigma due to COVID-19, loss of a significant one due to COVID-19, GAD-7 interpretation for anxiety were associated with depression category based on PHQ-9 interpretation (p<0.05; Table 2).

**Table 2:** Cross-tabulation of independent variables across anxiety category using Chi-square test *Fisher’s exact test employed.

Variables		PHQ-9 based diagnosis of depression			p-value
		No n(%)	Yes n(%)	Total n(%)	
Age (in years)	Less than 30	64(52.89)	57(47.11)	121(100.00)	0.049
	30 and above	61(66.30)	31(33.70)	92(100.00)	
Gender	Female	53(49.53)	54(50.47)	107(100.00)	0.006
	Male	72(67.92)	34(32.08)	106(100.00)	
Type of health institute	Government	61(62.24)	37(37.76)	98(100.00)	0.330
	Private	64(55.65)	51(44.35)	115(100.00)	
Province	Bagmati Province	79(55.63)	63(44.37)	142(100.00)	0.097*
	Gandaki Province	4(57.14)	3(42.86)	7(100.00)	
	Karnali Province	4(100.00)	0(0.00)	4(100.00)	
	Lumbini Province	8(61.54)	5(38.46)	13(100.00)	
	Province no. 2 (Janakpur as territorial capital)	5(41.67)	7(58.33)	12(100.00)	
	Province no.1 (Biratnagar as territorial capital)	14(87.50)	2(12.50)	16(100.00)	
	Sudurpaschim Province	11(57.89)	8(42.11)	19(100.00)	
Education	Bachelor	60(54.55)	50(45.45)	110(100.00)	0.414*
	Intermediate	13(54.17)	11(45.83)	24(100.00)	
	Masters or above	48(64.86)	26(35.14)	74(100.00)	
	Secondary school level	4(80.00)	1(20.00)	5(100.00)	
Marital status	Married	61(62.24)	37(37.76)	98(100.00)	0.330
	Single	64(55.65)	51(44.35)	115(100.00)	
Chronic diseases	No	120(60.30)	79(39.70)	199(100.00)	0.071
	Yes	5(35.71)	9(64.29)	14(100.00)	
History of psychiatric illness	No	121(59.61)	82(40.39)	203(100.00)	0.324*
	Yes	4(40.00)	6(60.00)	10(100.00)	
Psychiatric support in pandemic	No	114(60.32)	75(39.68)	189(100.00)	0.175
	Yes	11(45.83)	13(54.17)	24(100.00)	
Chronic diseases in family members	No	58(60.42)	38(39.58)	96(100.00)	0.642
	Yes	67(57.26)	50(42.74)	117(100.00)	
Living with the elderly (>60 years)	No	66(65.35)	35(34.65)	101(100.00)	0.061
	Yes	59(52.68)	53(47.32)	112(100.00)	
Medication for psychiatric illness	No	119(60.41)	78(39.59)	197(100.00)	0.074
	Yes	6(37.50)	10(62.50)	16(100.00)	
Frontline worker	No	29(65.91)	15(34.09)	44(100.00)	0.275
	Yes	96(56.80)	73(43.20)	169(100.00)	
Stigma due to COVID-19	No	61(68.54)	28(31.46)	89(100.00)	0.008
	Yes	58(50.00)	58(50.00)	116(100.00)	
Work experience	Less than five years	69(53.49)	60(46.51)	129(100.00)	0.056
	More than five years	56(66.67)	28(33.33)	84(100.00)	
Precautionary measures	Insufficient	86(60.99)	55(39.01)	141(100.00)	0.339
	Sufficient	39(54.17)	33(45.83)	72(100.00)	
COVID-19 diagnosed	No	101(62.35)	61(37.65)	162(100.00)	0.053
	Yes	24(47.06)	27(52.94)	51(100.00)	
Admitted to the hospital due to COVID-19	No	123(60.00)	82(40.00)	205(100.00)	0.068*
	Yes	2(25.00)	6(75.00)	8(100.00)	
Loss a significant one due to COVID-19	No	116(61.05)	74(38.95)	190(100.00)	0.044
	Yes	9(39.13)	14(60.87)	23(100.00)	
GAD-7 interpretation	No anxiety	105(92.92)	8(7.08)	113(100.00)	0.000*
	Mild anxiety	20(24.69)	61(75.31)	81(100.00)	
	Moderate anxiety	0(0.00)	17(100.00)	17(100.00)	
	Moderately severe anxiety	0(0.00)	2(100.00)	2(100.00)	
Anxiety	No	105(92.92)	8(7.08)	113(100.00)	0.000
	Yes	20(20.00)	80(80.00)	100(100.00)	
Healthcare workers	Other than doctor	50(53.19)	44(46.81)	94(100.00)	0.148
	Doctor	75(63.03)	44(36.97)	119(100.00)	

**Figure 3:**
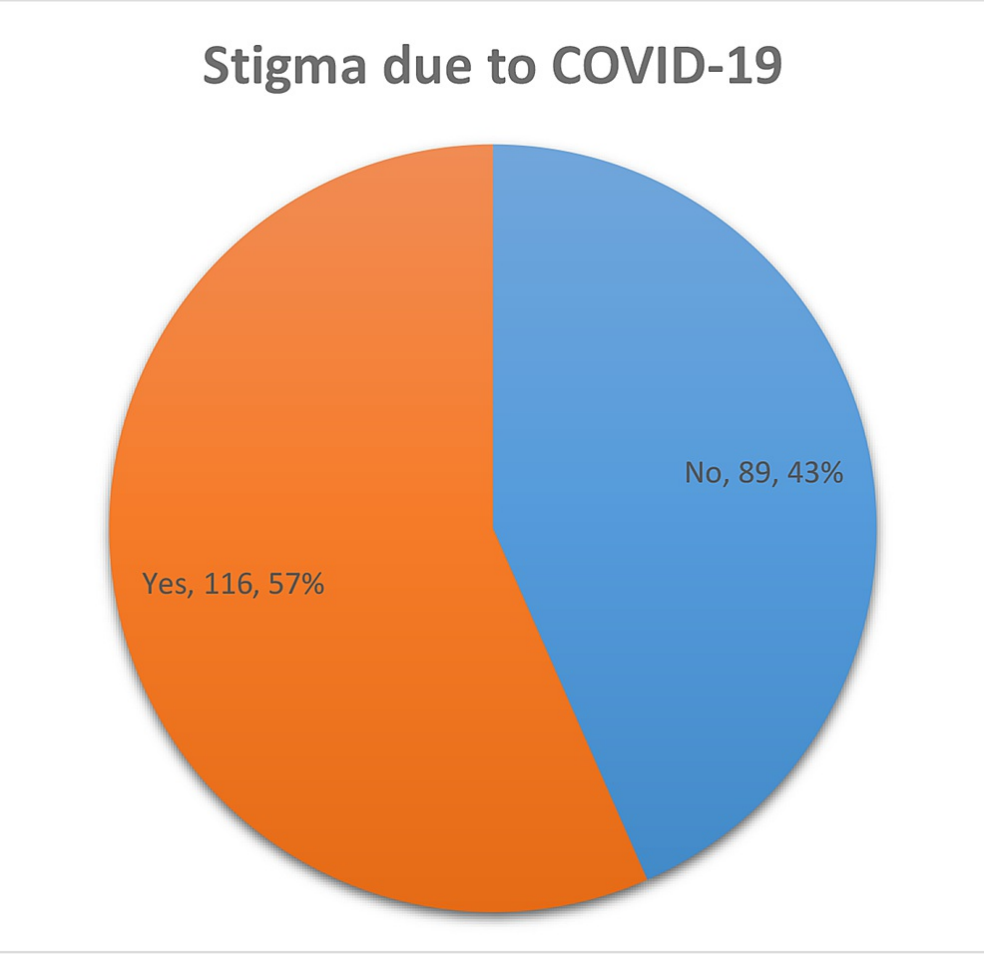
Stigma faced due to COVID-19 by healthcare practitioners

Stigmatization to the general public and healthcare practitioners was highly prevalent during the initial surge and mid-phase of the pandemic; 3.76% (n=8) did not want to mention their stigma. Among 205 respondents who disclosed their stigma status, 57% (n=116) faced some form of stigma in society due to COVID-19 (Figure 3).

Among categorical independent variables observed, living with elderly (>60 years), frontline working status, precautionary measures availability, COVID-19 diagnosed, GAD-7 interpretation for anxiety, and PHQ-9 interpretation for depression were associated with stigma category reported by respondents (p<0.05; Table 3).

**Table 3:** Cross-tabulation of independent variables across stigma category using Chi-square test *Fisher’s exact test employed.

Variables		Stigma due to COVID-19			p-value
		No n(%)	Yes n(%)	Total n(%)	
Age (in years)	Less than 30	50(42.74)	67(57.26)	117(100.00)	0.821
	30 and above	39(44.32)	49(55.68)	88(100.00)	
Gender	Female	43(42.16)	59(57.84)	102(100.00)	0.718
	Male	46(44.66)	57(55.34)	103(100.00)	
Type of institute	Government	35(36.84)	60(63.16)	95(100.00)	0.078
	Private	54(49.09)	56(50.91)	110(100.00)	
Province	Bagmati Province	58(42.34)	79(57.66)	137(100.00)	0.076*
	Gandaki Province	3(42.86)	4(57.14)	7(100.00)	
	Karnali Province	1(25.00)	3(75.00)	4(100.00)	
	Lumbini Province	3(23.08)	10(76.92)	13(100.00)	
	Province no. 2 (Janakpur as territorial capital)	4(36.36)	7(63.64)	11(100.00)	
	Province no.1 (Biratnagar as territorial capital)	12(80.00)	3(20.00)	15(100.00)	
	Sudurpaschim Province	8(44.44)	10(55.56)	18(100.00)	
Education	Bachelor	48(45.71)	57(54.29)	105(100.00)	0.832*
	Intermediate	9(39.13)	14(60.87)	23(100.00)	
	Masters or above	31(42.47)	42(57.53)	73(100.00)	
	Secondary school level	1(25.00)	3(75.00)	4(100.00)	
Marital status	Married	38(40.43)	56(59.57)	94(100.00)	0.427
	Single	51(45.95)	60(54.05)	111(100.00)	
Chronic diseases	No	85(44.27)	107(55.73)	192(100.00)	0.399*
	Yes	4(30.77)	9(69.23)	13(100.00)	
History of psychiatric illness	No	87(44.62)	108(55.38)	195(100.00)	0.192*
	Yes	2(20.00)	8(80.00)	10(100.00)	
Psychiatric support in pandemic	No	82(44.81)	101(55.19)	183(100.00)	0.245
	Yes	7(31.82)	15(68.18)	22(100.00)	
Chronic diseases in family members	No	38(41.76)	53(58.24)	91(100.00)	0.669
	Yes	51(44.74)	63(55.26)	114(100.00)	
Living with the elderly (>60 yrs)	No	49(51.04)	47(48.96)	96(100.00)	0.039
	Yes	40(36.70)	69(63.30)	109(100.00)	
Medication for psychiatric illness	No	82(43.39)	107(56.61)	189(100.00)	0.978
	Yes	7(43.75)	9(56.25)	16(100.00)	
Frontline worker	No	34(77.27)	10(22.73)	44(100.00)	0.000
	Yes	55(34.16)	106(65.84)	161(100.00)	
Work experience	Less than five years	56(45.16)	68(54.84)	124(100.00)	0.532
	More than five years	33(40.74)	48(59.26)	81(100.00)	
Precautionary measures	Insufficient	52(37.96)	85(62.04)	137(100.00)	0.025
	Sufficient	37(54.41)	31(45.59)	68(100.00)	
COVID-19 diagnosed	No	78(50.00)	78(50.00)	156(100.00)	0.001
	Yes	11(22.45)	38(77.55)	49(100.00)	
Admitted to the hospital due to COVID-19	No	88(44.67)	109(55.33)	197(100.00)	0.141*
	Yes	1(12.50)	7(87.50)	8(100.00)	
Loss of a significant one due to COVID-19	No	80(43.96)	102(56.04)	182(100.00)	0.660
	Yes	9(39.13)	14(60.87)	23(100.00)	
GAD-7 interpretation	No anxiety	54 (50.00)	54 (50.00)	108(100.00)	0.000*
	Mild anxiety	30 (37.97)	49 (62.03)	79(100.00)	
	Moderate anxiety	5 (31.25)	11 (68.75)	16(100.00)	
	Severe anxiety	0 (0.00)	2 (100.00)	2(100.00)	
PHQ-9 interpretation	No depression	61(51.26)	58(48.74)	119(100.00)	0.042*
	Mild depression	23(35.38)	42(64.62)	65(100.00)	
	Moderate depression	5(29.41)	12(70.59)	17(100.00)	
	Moderately severe depression	0(0.00)	3(100.00)	3(100.00)	
	Severe depression	0(0.00)	1(100.00)	1(100.00)	
Anxiety	No	54(50.00)	54(50.00)	108(100.00)	0.045
	Yes	35(36.08)	62(63.92)	97(100.00)	
Depression	No	61(51.26)	58(48.74)	119(100.00)	0.008
	Yes	28(32.56)	58(67.44)	86(100.00)	
Healthcare worker	Other than doctors	42(48.28)	45(51.72)	87(100.00)	0.228
	Doctor	47(39.83)	71(60.17)	118(100.00)	

GAD-7 scale and PHQ-9 scale questionnaire response

To gauge anxiety among respondents, we used GAD-7 standard questionnaire. The consistency of the scale was tested by Cronbach's alpha which showed high internal consistency of the scale for this study (number of items in the scale: 7; scale reliability coefficient: 0.8574). Similarly, PHQ-9 was used for depression evaluation. Cronbach's alpha coefficient for PHQ-9 was 0.8721 (number of items in the scale: 9) suggesting high internal consistency of the scale. The mean GAD-7 scale score among respondents was 4.72±3.42 (range, 0-16), and the mean PHQ-9 scale score was 4.51±3.87 (range, 0-21). Response to individual scale questionnaire over last two weeks at the time of the survey was presented in Table 4.

**Table 4:** Tabulation of GAD-7 and PHQ-9 response among respondents

GAD-7 scale questions					
Over the last two weeks, how often have you been bothered by the following problems?	Never (0) n(%)	Sometimes (1) n(%)	Frequently (2) n(%)	Always (3) n(%)	Total n(%)
Feeling nervous, anxious, or on edge	72(33.8)	124(58.2)	16(7.5)	1(0.5)	213(100.0)
Not being able to stop or control worrying	95(44.6)	97(45.5)	21(9.9)	0(0)	213(100.0)
Worrying too much about different things	81(38.0)	99(46.5)	32(15.0)	1(0.5)	213(100.0)
Trouble relaxing	84(39.4)	110(51.6)	18(8.5)	1(0.5)	213(100.0)
Being so restless that it’s hard to sit still	141(66.2)	62(29.1)	8(3.8)	2(0.9)	213(100.0)
Becoming easily annoyed or irritable	78(36.6)	107(50.2)	23(10.8)	5(2.3)	213(100.0)
Feeling afraid as if something awful might happen	98(46.0)	92(43.2)	20(9.4)	3(1.4)	213(100.0)
GAD-7 scale score: m±SD, 4.72±3.42; median, 4; range, 0-16					
PHQ-9 scale questions					
Little interest or pleasure in doing things	100(46.9)	97(45.5)	15(7.0)	1(0.5)	213(100.0)
Feeling down, depressed, or hopeless	117(54.9)	90(42.3)	6(2.8)	0(0)	213(100.0)
Trouble falling or staying asleep, or sleeping too much	96(45.1)	100(46.9)	15(7.0)	2(0.9)	213(100.0)
Feeling tired or having little energy	73(34.3)	114(53.5)	22(10.3)	4(1.9)	213(100.0)
Poor appetite or overeating	124(58.2)	69(32.4)	19(8.9)	1(0.5)	213(100.0)
Feeling bad about yourself or that you are a failure or have let yourself or your family down	136(63.8)	65(30.5)	11(5.2)	1(0.5)	213(100.0)
Trouble concentrating on things, such as reading the newspaper or watching television	119(55.9)	79(37.1)	13(6.1)	2(0.9)	213(100.0)
Moving or speaking so slowly that other people could have noticed. Or the opposite being so fidgety or restless that you have been moving around a lot more than usual	154(72.3)	54(25.4)	5(2.3)	0(0)	213(100.0)
Thoughts that you would be better off dead, or of hurting yourself	169(79.3)	41(19.2)	2(.9)	1(0.5)	213(100.0)
PHQ—9 scale score: Mean ±SD, 4.51±3.87; Median, 4; Range, 0-21					

Logistic regression

Logistic regression analysis was performed among dependent and independent variables. Only those variables where the association was seen in cross-tabulation were taken for logistic regression analysis. Multinomial logistic regression analysis showed significant odds of GAD-7 score interpretation suggesting anxiety among those with PHQ-9 interpretation of depression and vice versa (Tables 5 and 6). Similarly, HCWs working as frontline workers had significant odds of stigma compared to those not working in frontline (aOR, 6.48; CI, 2.84-14.80; P<0.001). Diagnosis of COVID-19 was associated with 3.09 times higher odds of facing stigma (CI, 1.37-6.98; P=0.007; Table 7).

**Table 5:** Logistic regression of GAD-7-based diagnosis of anxiety ®Reference taken.

Independent variables		Unadjusted				Adjusted			
		OR	[95% Conf. Interval]		p-Value	aOR	[95% Conf. Interval]		p-Value
Age (in years)	Age less than 30®								
	Age 30 and above in years	0.488506	0.28063	0.850365	0.011	0.785868	0.175624	3.516542	0.753
Gender	Female®								
	Male	0.474748	0.274531	0.820984	0.008	0.828374	0.304534	2.253292	0.712
Education	Secondary school level®								
	Bachelor	4.8	0.519651	44.33743	0.167	3.729472	0.092102	151.0162	0.486
	Intermediate	4.727273	0.458207	48.77079	0.192	2.990757	0.063162	141.6146	0.578
	Masters or above	2.166667	0.230043	20.40678	0.499	1.826325	0.038892	85.76193	0.759
Stigma due to COVID-19	No®								
	Yes	1.771429	1.011503	3.102274	0.046	1.046494	0.432233	2.533699	0.92
Work experience	Less than five years®								
	More than five years	0.552036	0.315388	0.96625	0.038	0.934223	0.254221	3.433119	0.918
Admitted to the hospital due to COVID-19	No®								
	Yes	8.430108	1.018729	69.76021	0.048	7.365117	0.459773	117.9821	0.158
PHQ-9 interpretation	No depression®								
	Depression	52.5	21.99548	125.3099	0.00	52.04255	20.15987	134.3474	0.00
Healthcare workers	Other than doctor®								
	Doctor	0.546046	0.315911	0.943828	0.03	0.723271	0.216058	2.421209	0.599

**Table 6:** Logistic regression PHQ-9-based diagnosis of depression ®Reference taken.

Independent variables		Unadjusted				Adjusted			
		OR	[95% Conf. Interval]		p-Value	aOR	[95% Conf. Interval]		p-Value
Age (in years)	Age less than 30®								
	Age 30 and above in years	0.5706068	0.3257295	0.999578	0.05	1.065827	0.4325134	2.62648	0.89
Gender	Female®								
	Male	0.4634774	0.2656077	0.808754	0.007	0.6946287	0.2932127	1.645594	0.408
Stigma due to COVID-19	No®								
	Yes	2.178571	1.223756	3.878364	0.008	2.10643	0.9006764	4.926352	0.086
Loss of a significant one due to COVID-19	No®								
	Yes	2.438438	1.004663	5.918382	0.049	1.747252	0.477902	6.388109	0.399
GAD-7 interpretation	No®								
	Yes	52.49997	21.99547	125.3098	0.00	48.20352	19.40214	119.7589	0.00

**Table 7:** Logistic regression for stigma due to COVID-19 ®Reference taken.

Independent variables		Unadjusted				Adjusted			
		OR	[95% Conf. Interval]		p-Value	aOR	[95% Conf. Interval]		p-Value
Living with the elderly (>60 yrs)	No®								
	Yes	1.798404	1.028883	3.143466	0.039	1.727965	0.9206344	3.243268	0.089
Frontline worker	No®								
	Yes	6.552725	3.013831	14.24705	0.00	6.483736	2.839423	14.80541	0.00
Precautionary measures	Insufficient®								
	Sufficient	0.5125596	0.2844371	0.92364	0.026	0.5857172	0.2995082	1.145427	0.118
COVID-19 diagnosed	No®								
	Yes	3.454545	1.646862	7.24644	0.001	3.091439	1.369583	6.978034	0.007
GAD-7 interpretation	No®								
	Yes	1.771429	1.011503	3.102274	0.046	1.0345	0.4215704	2.53858	0.941
PHQ-9 interpretation	No depression®								
	Depression	2.178571	1.223756	3.878364	0.008	1.941687	0.7698868	4.897017	0.16

Relation among continuous variables

**Figure 4:**
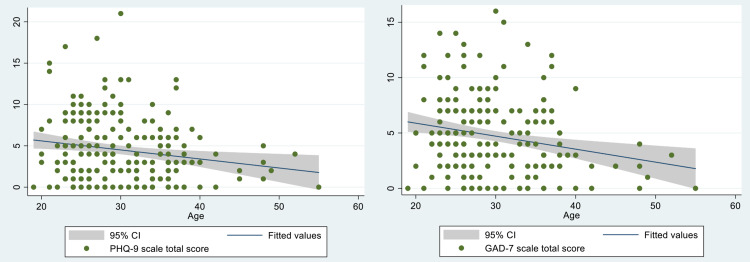
Correlation between the age of the participants and scores (A) Correlation of PHQ-9 score with age and (B) correlation of GAD-7 score with age.

The relation between age and PHQ-9 and GAD-7 total score was evaluated by plotting a scatter plot. It showed a weak negative correlation between the age of the participants and scores (co-efficient for PHQ-9 score: −0.1811, and for GAD-7 score: −0.2201; Figure 4A and 4B). Every one-year increment in age showed 0.4133047 times decrement in GAD-7 score (CI, −0.661881 to −0.1647284; p=0.001). Similarly, one-year increment in age showed 0.3002436 times decrement in PHQ-9 score (CI, −0.5215687 to −0.0789186; p=0.008).

## Discussion

Multiple studies have evaluated the impact of COVID-19 among healthcare professionals during the early stages of the pandemic in Nepal. We aimed to assess the mental health impact in the late phase of the COVID-19 pandemic because longitudinal analysis across a time period is essential to gauge the long-term effects on HCWs who act as a frontline defense against the pandemic. We found that the prevalence of anxiety and depression was 46.95% and 41.31% among healthcare professionals. Our findings were higher compared to the earlier studies done in Nepal among healthcare professionals. A study by Khanal et al. found that 41.9% of health workers had symptoms of anxiety and 37.5% had depressive symptoms while Pandey et al. reported the symptoms of anxiety and depression were present among 35.6% and 17.0% of HCWs, respectively [6,11]. Gupta et al. too reported that the prevalence of anxiety disorder was 37.3% among HCWs, with the majority of the participants having mild anxiety and 8% of the participants had depression [7]. Most of these studies were conducted from April to May 2020 at the beginning of the pandemic during which there were no mortalities and severe forms of the disease in Nepal [6,7,11]. The first mortality due to COVID-19 in Nepal was reported only in May 2020 and maximum mortalities due to COVID-19 occurred from October to December 2020 as per John Hopkins data for COVID-19 [12]. The increased prevalence seen in our study might be due to the time of our study. Our findings are significant because it highlights the mental distress evident in the health care workers even in the later phase of the first wave of the pandemic despite vaccination and therapeutics (like repurposing of drugs already used in other condition or new experimental agents) to combat the pandemic. The ever-growing news about the new B.1.351 variant and B-117 variant of COVID-19 might have also contributed to our findings because the newer strains have been found to spread more rapidly and the Astrazeneca vaccines are less efficacious especially against the South African variant [13,14]. The culmination of these events might have led to the distress because most HCWs now realize that the pandemic may last longer than previously anticipated. HCWs are more vulnerable than the general population to develop abnormal mental disorders and symptoms due to the increased risk of exposure to infected patients.

We found that age, gender, education level, stigma due to COVID-19, work experience, hospital admission due to COVID-19, PHQ-9 interpretation for depression, and health practitioner category were significantly associated with provoking/preventing anxiety disorder based on GAD-7 by running the Pearson Chi-square test and unadjusted logistic regression analysis. It is important to note that women have been found to experience more distress and anxiety compared to males in multiple studies [11,15]. Women are usually the caregivers in the family and the professional burden coupled with responsibility and social norms might lead to excessive distress and anxiety. Also, a meta-analysis by Sanghera et al. showed that less working experience was associated with worse mental outcomes among the eight included studies in the analysis [16]. Health personnel has been found to have more distress compared to the general population as per many studies done worldwide and in Nepal [6,11,15]. However, adjusting across the variables and running multinomial logistic regression analysis showed the relation holds true only for PHQ-9 interpretation for depression and anxiety disorder based on GAD-7.

We also found stigma due to COVID-19, loss of a significant one due to COVID-19, GAD-7 interpretation for anxiety were associated with depression. Teksin et al. found a statistically significant positive correlation between the perception of stigmatization score and HAD-S (Hospital Anxiety Depression Score) [17]. Also, Teksin et al. reported a statistically significant negative correlation between the perception of the stigmatization score and the Psychological Well-Being Score, Coping Styles Scale brief form (CBSS-BF) problem-focused coping and emotion-focused coping, and all subscales of World Health Organization Quality of Life Scale short form (WHO-QOL BREF) [17]. Thus, stigmatization is associated with poor quality of life and adverse mental outcomes like depression. Anxiety and depression have been found to exist as comorbid conditions together accounting for 23.2% in the study by Sigdel et al. [18]. This could be due to the psychological impact caused by the COVID-19 pandemic putting a mental and physical burden among healthcare professionals in addition to the increased fear of contracting the virus. This explains the association between GAD-7 interpretation of anxiety and depression category based on PHQ-9 interpretation given their presence as co-morbid conditions in the heat of the pandemic.

We found that frontline working status was associated with stigma such that frontline workers were three times more likely to experience stigma compared to those not working in the frontline. Infectious disease outbreaks have been found to cause stigma among HCWs since the past [19]. Similarly, a study in Turkey found that HCWs who had worked with COVID-19 patients with less training were found to experience more stigma [17]. Zandifar et al. reported that working in the frontline increased the odds of intrusion and hypervigilance in a study done in Iran [20]. This might explain the association between the working status of the frontline worker and stigma. Duy et al. found a moderate correlation between the stigma scale and 21‐item Depression, Anxiety, and Stress Scale subscale scores [21]. This finding was similar to our finding of an association of GAD-7 interpretation for anxiety and PHQ-9 interpretation for depression with stigma. Also, the diagnosis of COVID-19 among HCWs was found to be significantly associated with stigma. Stigmatization has been found among COVID-19 survivors in a study done in India and Teksin et al. reported an increased association of stigma with HCWs who experienced COVID-19 symptoms themselves [17,22]. The fear of being infected with coronavirus and the unpredictable clinical sequelae of infection might explain why HCWs with a diagnosis of COVID-19 might likely experience stigma. We also found that living with the elderly and the availability of precautionary measures to be associated with stigma. HCWs feel protected with sufficient precautionary measures leading to a feeling of self-assurance and protection for infection with COVID-19. Decreased fear of being infected with COVID-19 with precautionary measures may lead to decreased odds of stigma because HCWs without COVID-19 do not face the same level of stigma as healthcare workers who are infected with COVID-19.

Our study has several limitations. First, the findings of our study cannot be generalized to the whole population as it is focused on HCWs. Our study is a cross-sectional study with a small sample size. There are no validated tools to assess COVID-19 related stigma, and thus, the perception of stigma experienced by health care workers was reported by individual respondents. Since our survey was web-based with only an English version of the questionnaire with the assumption of adequate education and understanding of the participants, it could have limited access and understanding to some HCWs who did not have access to the internet and limited education. Additionally, we have used PHQ-9 directly for screening depression as it is a well-validated tool to make our study simple instead of using PHQ-2 following the use of PHQ-9, which could be another limitation.

## Conclusions

The prevalence of anxiety and depression was significant among healthcare professionals. There was a bidirectional relationship between GAD-7-based diagnosis of anxiety and PHQ-9 score interpretation. More than half of HCWs faced some form of stigma in society due to COVID-19. HCWs working as frontline workers and those with a diagnosis of COVID-19 have increased odds of stigma.
